# Comparative Study of Δ9-Tetrahydrocannabinol and Cannabidiol on Melanogenesis in Human Epidermal Melanocytes from Different Pigmentation Phototypes: A Pilot Study

**DOI:** 10.3390/jox12020012

**Published:** 2022-06-10

**Authors:** Shilpi Goenka

**Affiliations:** 1 Department of Biomedical Engineering, Stony Brook University, Stony Brook, NY 11794-5281, USA; shilpi.goenka@stonybrook.edu; 2 Department of Biochemistry and Cell Biology, Stony Brook University, Stony Brook, NY 11794-5281, USA

**Keywords:** Δ9-tetrahydrocannabinol, cannabidiol, human melanocytes, lightly-pigmented, darkly-pigmented, melanogenesis, dendricity, reactive oxygen species

## Abstract

Δ9-tetrahydrocannabinol (THC) is one of the primary ingredients of cannabis plants and is responsible for the psychoactive properties of cannabis. While cannabidiol (CBD), the non-psychoactive compound from cannabis, has been shown to stimulate human epidermal melanogenesis, the effects of THC have not been addressed in human epidermal melanocytes. Moreover, to date, no study has tested the effects of these compounds on melanocytes differing in pigmentation, representative of different skin phototypes, which would be significant as different ethnicities are known to differentially metabolize these xenobiotics. Herein, the effects of THC were studied and compared alongside CBD in human epidermal melanocytes derived from lightly-pigmented (HEMn-LP; Caucasian) and darkly-pigmented (HEMn-DP; African-American) cells over a chronic exposure of 6 d. Results demonstrated that both compounds displayed cytotoxicity at 4 µM but stimulated melanin synthesis and tyrosinase activity in a similar manner in LP and DP cells at nontoxic concentrations of 1–2 µM. However, THC and CBD showed a differential effect on dendricity in both cells; THC and CBD reversibly increased dendricity in LP cells while there was no significant change in DP cells. THC and CBD induced higher levels of reactive oxygen species (ROS) in LP cells while there was no change in the ROS levels in DP cells. In summary, although THC was relatively less cytotoxic as compared to CBD to both LP and DP cells, it exhibited a similar capacity as CBD to stimulate melanin synthesis and export in LP cells which was accompanied by a significant oxidative stress. DP cells were relatively resistant to the effects of both THC and CBD which might implicate the protective effects conferred by melanin in dark-skinned individuals.

## 1. Introduction

Marijuana (or cannabis) is an illicit controlled substance, the second most widely smoked drug after tobacco, and is a major public health concern [1,2]. THC (Δ9-tetrahydrocannabinol) is the primary psychoactive compound in cannabis while cannabidiol (CBD), another constituent of cannabis, is non-psychoactive [3]. The increasing legalization of cannabis [4] has made it largely available for medical and recreational purposes in the form of smoking, dabbing, edibles, topical ointments, or tinctures [5]. Joints for recreational use typically have a THC content of 7 mg [6]. The dried weight content of THC in skunk-type cannabis is higher than 20%, while non-skunk cannabis has lower THC contents ranging from 2–8% [7]. Smokers can increase the intake of THC via titration during smoking. Although THC concentrations in hemp-derived CBD are regulated in the USA to not exceed 0.3%, significantly higher levels of THC were detected in 21% of CBD oils which were originally marketed to have very low or undetectable THC concentrations [8] thus increasing the risk of THC exposure. In addition, a recent report [9] documented that CBD in e-cigarettes can transform into the psychoactive THC during evaporation, thus raising further concerns of higher exposures to these xenobiotics.

As the key specialized cells that originate from the neural crest, melanocytes play a critical role in the synthesis of the pigment melanin in the epidermis, iris, hair, ears, brain, and lungs [10,11]. Although melanocytes constitute a minority population in the suprabasal layers of the epidermis, they are capable of extending multiple dendrites that facilitate their contact with multiple keratinocytes; a single melanocyte can make contact with up to 40 keratinocytes [12,13]. Tyrosinase is one of the key enzymes which catalyzes the initial steps in biosynthesis of melanin [14]. The pigment melanin imparts photoprotective benefits and acts as a free-radical scavenger [15]. Abnormal accumulation of melanin in the skin is a risk factor for melanoma.

Previous reports have shown the differential impact of nicotine, the primary constituent of tobacco smoke, on melanogenesis in human melanocytes; nicotine was shown to suppress melanin synthesis and tyrosinase activity in HEMn-LP cells [16] while it enhanced melanin synthesis and tyrosinase activity in HEMn-DP cells [17]. Interestingly, in these studies, nicotine was shown to induce oxidative stress in cells from both pigmentation types. Tobacco smoking has also been shown to induce higher levels of melanin synthesis in primary melanocytes [18]. As compared to tobacco smoke, marijuana smoke is perceived to be less harmful [19] although it stays longer due to larger puffs, deep inhalation, and a fourfold higher breath hold time [20]. Primary human melanocytes possess an endocannabinoid system as well as their target G protein-coupled receptors CB_1_ and CB_2_. Furthermore, the endocannabinoid anandamide (AEA) was shown to stimulate melanin synthesis and tyrosinase activity [21]. However, in another study, endocannabinoids suppressed melanin synthesis in human melanoma cells and keratinocyte cocultures under basal and UV-irradiated conditions, and the effects were shown to be mediated by the CB1 receptor [22]. Interestingly, no study has examined the impact of THC that is known to exert its biological action by mimicking endocannabinoids. The paucity of studies on the effects of THC in normal human melanocytes prompted us to study whether THC, like nicotine, might impact melanogenesis and cause adverse effects. CBD, the non-psychoactive compound, was included for comparison in the study to identify if there may be any differences in the effects of these two compounds, especially since THC has previously been shown to diminish the melanin content of human hair follicles [23], whereas CBD was shown to stimulate the melanin content of human epidermal melanocytes [24]. It is of note that none of these reports evaluated the ethnicity-dependent differences which arise from different skin photo-types.

Cannabinoids are metabolized by hepatic microsomal cytochrome P450 (CYP450) enzymes; THC is primarily metabolized by isoenzymes CYP2C9 and CYP3A4 [25] while CBD is metabolized by CYP2C19 in addition to CYP3A4 [26]. Polymorphisms in CYP2C9 have been shown to result in different bioavailability of THC, causing higher chances of exposure to this xenobiotic [27]. As compared to other ethnicities, Caucasians have 35% higher prevalence rate of CYP2C9 polymorphisms that can suppress the metabolic activity of CYP2C9, which in turn enhances the bioavailability of THC by almost two- or threefold as shown in prior clinical studies [28,29]. Based on this, it is clear that the study of these xenobiotics in light and dark-skinned skin will be necessary to establish their biochemical effects in ethnically different populations. However, to date, there is a lack of any report where the effects of THC on melanogenesis in normal human melanocytes or their comparison with CBD were explored. Hence, this study sought to investigate and compare the effects of THC as well as CBD on melanogenesis and oxidative stress in vitro using HEMn-LP and HEMn-DP cells. Furthermore, the effects of compounds on melanin export were indirectly evaluated by quantifying melanocyte dendricity; this is critical as many compounds which increase melanin synthesis may inhibit melanosome export causing depigmentation. Hence, in order to confirm the pro-melanogenic activity, it is important to test the effects on both melanin synthesis and export.

## 2. Materials and Methods

### 2.1. Materials

THC and CBD were kindly provided by Dr Dale Deutsch (Department of Biochemistry and Cell Biology, Stony Brook University, NY, USA). A CellTiter 96^®^ AQueous One Solution Cell Proliferation Assay (MTS) assay was procured from the Promega Corporation (Madison, WI, USA). Medium 254 and human melanocyte growth supplement (HMGS) was procured from Cascade Biologics (Portland, OR, USA). A bicinchoninic acid (BCA) assay kit (Pierce™), Hanks balanced salt solution (HBSS), phosphate buffered saline (PBS) and penicillin-streptomycin mixture (Gibco™) were purchased from Thermo Scientific (Waltham, MA, USA). L-DOPA (3,4-Dihydroxy-L-phenylalanine; ≥98%) was purchased from Millipore Sigma (Burlington, MA, USA). DCFH-DA (2′-7′dichlorofluorescin diacetate) dye was procured from Molecular Probes (Invitrogen, CA, USA).

### 2.2. Cell Culture

Human epidermal melanocytes from neonatal darkly-pigmented (HEMn-DP) and lightly-pigmented (HEMn-LP) donors were obtained from Cascade Biologics (Portland, OR, USA) and maintained in Medium 254 supplemented with 1% HMGS and 1% penicillin-streptomycin antibiotic mixture. All cells were grown at 37 °C in a 95% air–5% CO_2_ humidified incubator.

### 2.3. Cytotoxicity Assay

HEMn-LP and HEMn-DP cells (3 × 10^4^ cells/well) were seeded in 24-well plates for 24 h followed by the addition of compounds in DMSO (final DMSO concentration in all groups including control was 0.4%) and incubated for a duration of 6 d (with compounds replenished on third day). After the 6 d treatment duration, the culture medium was replaced by 200 μL of fresh medium containing 40 μL of MTS dye and incubated for 1 h after which the absorbance of 100 μL aliquots was read at 490 nm using a Versamax^®^ microplate reader. Cell viability was calculated from the absorbance values relative to negative control groups and expressed in percentages.

### 2.4. Intracellular Melanin Assay

HEMn-LP cells (1.5 × 10^5^ cells/well) or HEMn-DP cells (1.2 × 10^5^ cells/well) were cultured in 6-well plates for 24 h followed by the addition of test compounds and further cultured for 6 d. The relative levels of cellular melanin were determined in a manner similar to the method reported in previous studies [30,31]. Briefly, the cells were harvested, washed in PBS, and 150 μL of 1 N NaOH was added and heated to 70 °C to solubilize melanin. Following this, 100 µL aliquots of lysate were transferred to a 96-well plate and the absorbance was read at 475 nm using a microplate reader. The absorbance values were normalized to the total protein contents; Abs/µg protein was expressed as percentage of control. The basal melanin content levels of LP and DP cells were already reported in our previous study where DP cells exhibited a 1.34-fold higher melanin content as compared to LP cells [32].

### 2.5. Intracellular Tyrosinase Activity

HEMn-LP cells (1.5 × 10^5^ cells/well) or HEMn-DP cells (1 × 10^5^ cells/well) were cultured in 6-well plates for 24 h followed by the addition of compounds, and further incubated for 6 d. At the end of treatments, the cells were detached and lysed; 25 µL of lysates was then combined with 75 µL of freshly prepared 3 mM L-DOPA in a 96-well microplate and the absorbance was measured at 475 nm for a period of 30 min at 30 °C in the kinetic mode using a microplate reader. The percentage of tyrosinase activity was calculated from the slope of the linear range of the velocities of inhibition and was normalized by the total protein content.

### 2.6. Measurement of Melanocyte Dendricity

HEM-LP cells were seeded in 12-well plates (2.5 × 10^4^) for 24 h followed by the replacement of the medium by fresh medium containing compounds, and the cultures were incubated for a period of 6 d (with one renewal in between). After the treatments, random microscopic fields in each well were imaged at 20× objective magnification. The images were analyzed using an NIS Elements computer-aided image analyzer and two parameters, (i) number of dendrites and (ii) total dendrite length, were quantified in a manner similar to the method reported in previous studies [30,33]. HEMn-DP (3.8 × 10^4^ cells/well) was seeded in a 6-well plate for 48 h and compounds was then added for 6 d, and similar measurements were performed.

### 2.7. Recovery Study of Dendricity

In order to test if the effects of compounds on dendricity were reversible upon withdrawal of compounds from the medium, 0.6 × 10^5^ LP cells were cultured in 35 mm petri dishes for 48 h followed by treatment with test compounds and cultures maintained for 6 d, after which the cells were imaged for exposure groups. At this point, the wells were washed in HBSS, and the culture medium was renewed with fresh medium without compounds and maintained for a period of 6 d. At the end of 6 d recovery period, the cells were imaged, and dendricity was quantified similar to the method reported described earlier.

### 2.8. Intracellular Reactive Oxygen Species (ROS)

Intracellular ROS generation was quantified using DCFH-DA, that is, a cell-permeable and non-fluorescent probe which is cleaved by cellular esterases to H2DCF that is then further oxidized by ROS to fluorescent 2′, 7′-dichlorofluorescein (DCF). A quantity of 6 × 10^4^ HEMn-LP cells/well or 4 × 10^4^ HEMn-DP cells/well were cultured in 12-well plates for 24 h, followed by the replacement of the medium with test compounds and cultures maintained for a period of 6 d (with compound renewal once). At the end of the incubation period, cells were washed in HBSS and incubated in 50 µM DCFH-DA probe solution and incubated at 37 °C for 45 min. After this step, cells were washed, lysed, and centrifuged. The supernatants were aliquoted in a 96-well black plate (Greiner) and fluorescence was recorded at an excitation/emission wavelength of 485/535 nm using a fluorescence microplate reader (Gemini EM Spectramax, Molecular Devices). The relative fluorescence intensity (RFU) values were normalized to total protein content and expressed as percentage of an untreated control in a manner similar to a method reported previously [34].

### 2.9. Statistical Analysis

One-way analysis of variance (ANOVA) with Tukey’s post-hoc test was run using GraphPad Prism software. Differences were considered statistically significant at *p* < 0.05. All data are reported as mean ± SD.

## 3. Results

### 3.1. Effects of Compounds on Viability of HEMn-LP and HEMn-DP Cells

**Figure 1:**
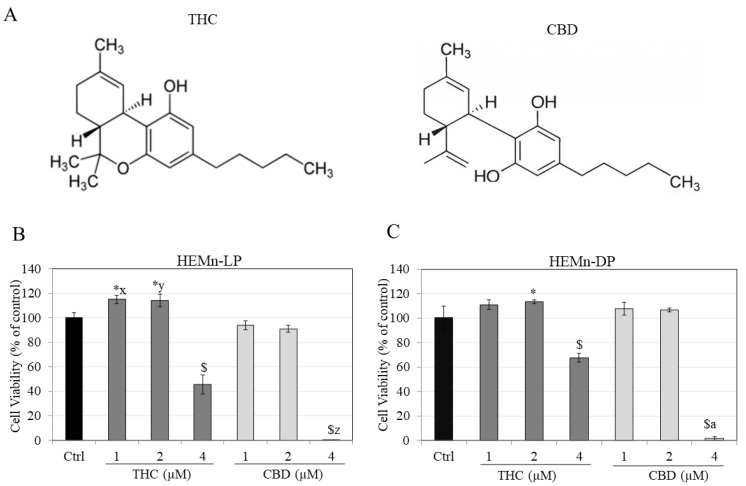
(**A**) Chemical structures of THC and CBD; viabilities of (**B**) HEMn-LP cells and (**C**) HEMn-DP cells treated with THC and CBD for a period of 6 d. All data are mean ± SD of triplicates. One-way ANOVA followed by Tukey’s test; [* *p* < 0.05 and $ *p* < 0.001 vs. Ctrl; letter x: *p* < 0.001 vs. CBD (1 µM), letter y: *p* < 0.01 vs. CBD (2 µM), letter z: *p* < 0001 vs. THC (4 µM) and letter a: *p* < 0.001 vs. THC (4 µM)].

The chemical structures of THC and CBD, which are structural isomers, is illustrated in Figure 1A. THC and CBD were first assessed for cytotoxicity to both cell types in order to identify nontoxic concentration ranges to be used for subsequent experiments.

Results showed that THC caused significant cytotoxicity to HEMn-LP cells at the highest concentration of 4 µM, where the viability was reduced by 54.47%, while at lower concentrations, it significantly enhanced viability by 15.09% and 14.27% at 1 and 2 µM, respectively (Figure 1B). On the other hand, CBD induced higher cytotoxicity to HEMn-LP cells at 4 µM with ~100% cell loss which was significantly higher as compared to cell loss at THC (4 µM). There was no change in the viability of cells by CBD at concentrations lower than 4 µM (Figure 1B).

In HEMn-DP cells, THC significantly lowered viability by 32.60% at 4 µM, while at lower concentration of 2 µM, there was an increase in viability by 13% (Figure 1C). CBD induced higher cytotoxicity to DP cells at 4 µM, with no effect at lower concentrations (Figure 1C). Altogether, these results show that THC is less cytotoxic to both LP and DP cells as compared to CBD. Based on these results, THC and CBD were selected at concentrations of 1 and 2 µM for subsequent experiments.

### 3.2. Effects of Compounds on Melanin Synthesis, Tyrosinase Activity and Melanocyte Dendricity in HEMn-LP Cells

**Figure 2:**
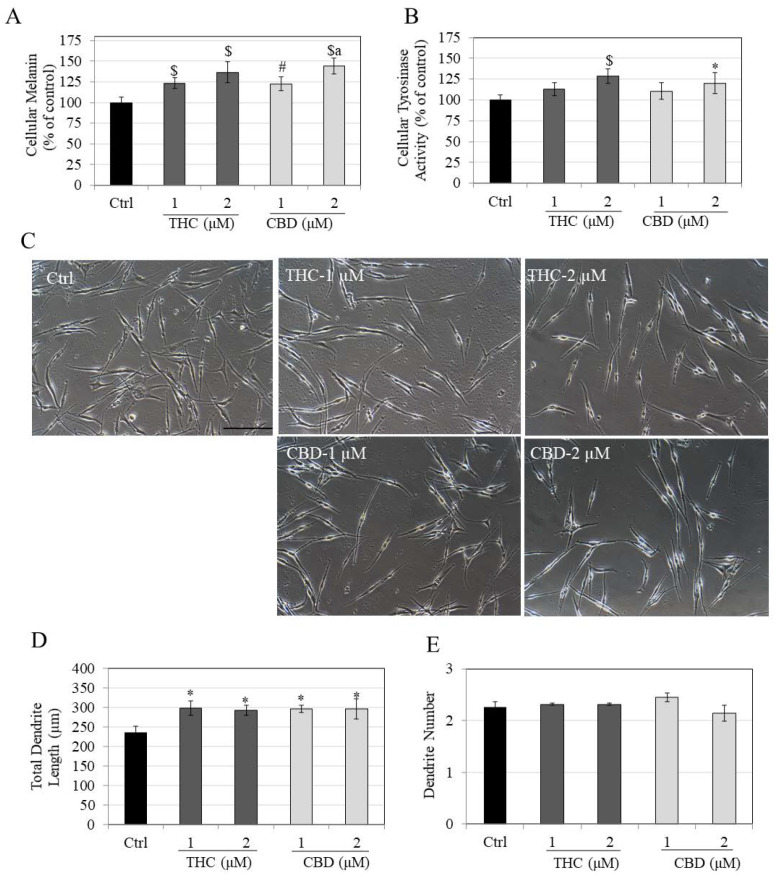
(**A**) Intracellular melanin levels; (**B**) Intracellular tyrosinase activity in HEMn-LP cells treated with THC and CBD for 6 d; (**C**) Representative phase-contrast images (20× magnification) of LP cells for control group and groups treated with THC and CBD at 1 and 2 µM; the two tail-like cytoplasmic extensions of LP cells are considered its two dendrites; (**D**) Total dendrite length; and (**E**) Dendrite number. A total of ~100 cells were analyzed for each treatment group from triplicate wells. Data for (**A**,**B**) are mean ± SD of values combined from two independent experiments; one-way ANOVA followed by Tukey’s test; [* *p* < 0.05 vs. Ctrl; # *p* < 0.01 vs. Ctrl; $ *p* < 0.001 vs. Ctrl; letter a: *p* < 0.01 vs. CBD (1 µM)].

Both THC and CBD stimulated melanin synthesis in HEMn-LP cells in a concentration-dependent manner. THC significantly stimulated melanin synthesis by 23.52% and 36.71% at concentrations of 1 µM and 2 µM, respectively (Figure 2A). CBD showed a similar trend with a significant increase of 22.76% and 44.29% at 1 µM and 2 µM, respectively. Furthermore, the increase in melanin synthesis by CBD at 2 µM was significantly higher by 21.53% than levels at 1 µM.

Subsequently, the intracellular tyrosinase activities were determined to examine if the stimulation of melanin synthesis might be correlated to the stimulation of tyrosinase activity. The intracellular tyrosinase activities in THC-treated cells showed a marginal increase of 12.91% at 1 µM, but a significant increase of 28.59% at 2 µM (Figure 2B) as compared to control. CBD showed a similar result as that of THC with a significant increase of 20.35% at 2 µM. These results show that the increase in melanin synthesis by THC and CBD can be explained, at least in part, by the increase in tyrosinase activity.

The significance of melanin export as a further step in the melanogenesis cycle in melanocytes has been well established; hence, THC and CBD were further examined for any effects on melanin export, which was estimated by quantitating the total dendrite length (TDL) and number of dendrites. The images of cells showed a visible elongation in dendrites treated with both THC and CBD at 1 µM and 2 µM in a similar fashion (Figure 2C). Quantitation of dendritic parameters further demonstrated that THC significantly increased TDL by 25.71% and 23.71% at 1 µM and 2 µM, respectively, as compared to control (Figure 2D). CBD showed very similar effects on TDL as that of THC with significant increases of 25.02% at 1 µM and 25.25% at 2 µM. However, none of the compounds had any effect on dendrite numbers at any concentration (Figure 2E).

Collectively, the results showed that in HEMn-LP cells, THC, and CBD displayed similar effects in stimulating melanin synthesis as well as dendricity over the concentration range 1–2 µM.

### 3.3. Effects of Compounds on Melanin Synthesis, Tyrosinase Activity and Melanocyte Dendricity in HEMn-DP Cells

**Figure 3:**
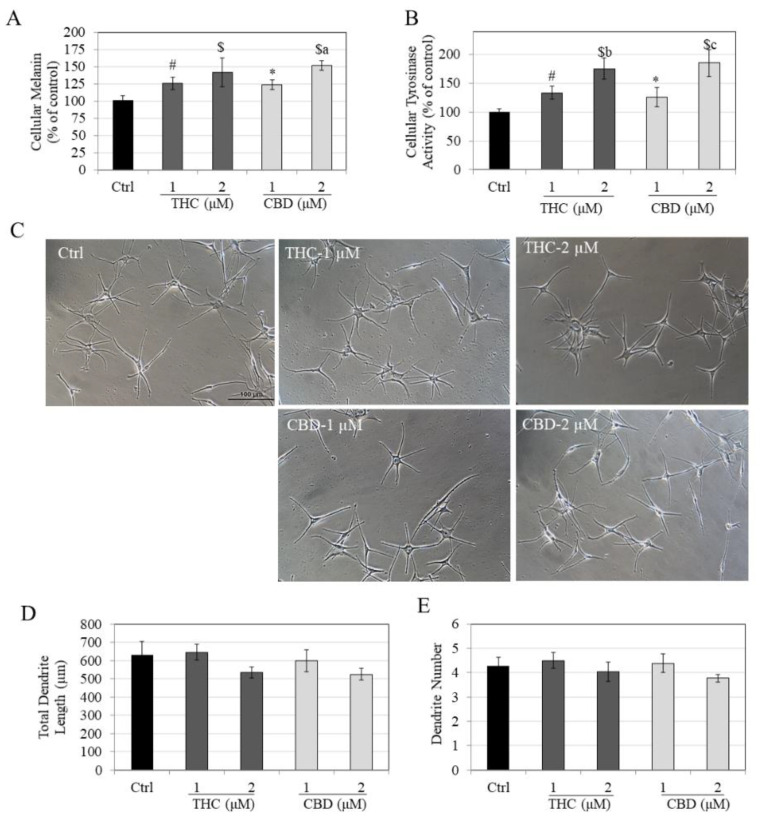
(**A**) Intracellular melanin levels; (**B**) Intracellular tyrosinase activity in HEMn-DP cells treated with THC and CBD for 6 d; (**C**) Representative phase-contrast images (20× magnification) of DP cells for control group and groups treated with THC and CBD at 1 µM and 2 µM; (**D**) Total dendrite length; and (**E**) Dendrite number. A total of ~100 cells were analyzed for each treatment group from triplicate wells. Data for (**A**,**B**) are mean ± SD of values combined from two independent experiments; one-way ANOVA followed by Tukey’s test; [* *p* < 0.05 vs. Ctrl; # *p* < 0.01 vs. Ctrl; $ *p* < 0.001 vs. Ctrl; letter a: *p* < 0.01 vs. CBD (1 µM); letter b: *p* < 0.001 vs. THC (1 µM); letter c: *p* < 0.001 vs. CBD (1 µM)].

Similar to the results obtained earlier in LP cells, both THC and CBD dose-dependently stimulated melanin synthesis in HEMn-DP cells. THC significantly stimulated melanin synthesis by 25.53% and 41.58% at concentrations of 1 µM and 2 µM, respectively (Figure 3A). CBD significantly increased cellular melanin by 22.88% and 51.36% at 1 µM and 2 µM, respectively. Moreover, the increase in melanin synthesis by CBD at 2 µM was significantly higher than CBD at 1 µM by 28.48% (Figure 3A).

The intracellular tyrosinase activities in THC-treated cells showed a significant increase of 33.76% and 75.57% at concentrations of 1 µM and 2 µM, respectively (Figure 3B) as compared to control. The increase in tyrosinase activity by THC at 2 µM was significantly higher (41.81% higher) than levels at 1 µM. CBD showed a similar result of dose-dependent increase with a significant increase of 25.91% and 85.33% at concentrations of 1 µM and 2 µM, respectively. The increase in tyrosinase activity by CBD at 2 µM was significantly higher (59.41%) than levels at 1 µM. These results show that the increase in melanin synthesis by THC and CBD can be explained, at least in part, by the corresponding increase in tyrosinase activity.

The phase-contrast micrographs of HEMn-DP cells in the control group showed arborized morphology as expected for these cells; this morphology appeared to be retained in both THC- and CBD-treated groups, although the cells treated with both compounds at 2 µM seemed to show a reduced dendrite length (Figure 3C). Quantification of the dendritic parameters showed that THC (2 µM) and CBD (2 µM) reduced TDL by 15.14% and 16.65%, respectively, as compared to control, although no statistical significance was reached (Figure 3D). Furthermore, THC and CBD did not affect the number of dendrites of these cells at any concentration (Figure 3E).

Taken together, the results showed that in DP cells, THC and CBD displayed similar effects in stimulating melanin synthesis but showed a trend towards inhibiting melanin export at a concentration of 2 µM.

### 3.4. Effects of Compounds on Recovery of Melanocyte Dendricity in HEMn-LP Cells

**Figure 4:**
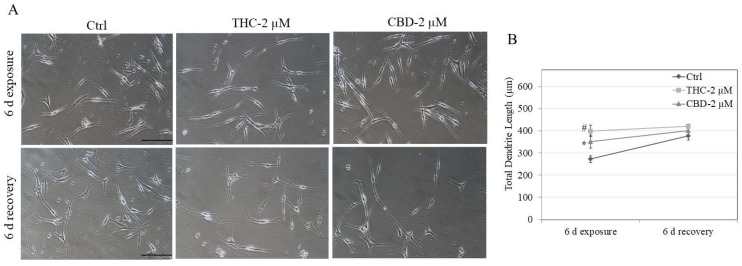
Recovery study of dendricity in HEMn-LP cells after washout of THC and CBD from the culture medium. (**A**) Representative phase-contrast micrographs showing HEMn-LP cells treated with THC and CBD at 2 µM for a period of 6 d followed by the removal of compounds from the culture medium and continuation of cultures in fresh medium for an additional period of 6 d; (**B**) Quantification of total dendrite length (TDL) of LP cells after the exposure period and after the recovery period. Data are mean ± SD of triplicates; a minimum of at least 100 cells were counted in each treatment (Ctrl, THC, CBD) for both exposure and recovery groups; # *p* < 0.01 and * *p* < 0.05 vs. Ctrl of 6 d exposure; one-way ANOVA with Tukey’s test.

Additional experiments were conducted to evaluate whether the stimulatory effects of THC and CBD on HEMn-LP cell dendrites might be reversed upon the removal of these xenobiotics from the culture medium. As both THC and CBD are structural isomers, it was of interest to examine whether there may be a differential recovery in dendricity. As shown in Figure 4A, both THC and CBD stimulated TDL significantly, which was also visible on micrographs, and after cessation of treatments with both compounds, the TDL completely recovered to control values at 6 d recovery (Figure 4B). These results indicated that the differences in structure do not lead to differential recovery and that both compounds exhibited similar recoveries.

### 3.5. Effects of Compounds on Intracellular ROS Generation in HEMn-LP and HEMn-DP Cells

**Figure 5:**
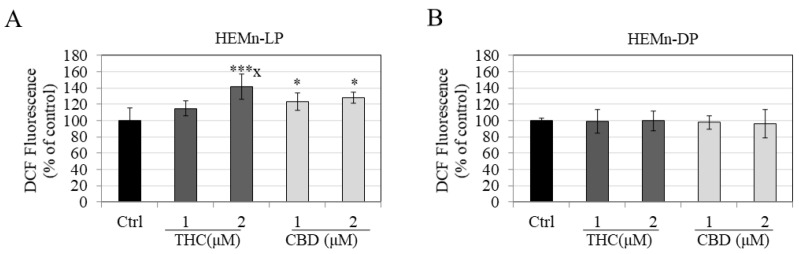
Determination of levels of intracellular ROS generation by DCFDA assay in (**A**) HEMn-LP cells and (**B**) HEMn-DP cells treated with THC and CBD at concentrations of 1 and 2 µM for a duration of 6 d. Data are mean ± SD of values combined from two independent experiments. * *p* < 0.05 vs. Ctrl; *** *p* < 0.001 vs. Ctrl; letter x: *p* < 0.05 vs. THC (1 µM); one-way ANOVA with Tukey’s test.

The effects of THC and CBD on oxidative stress in melanocytes were determined by the levels of ROS generation. Both THC and CBD increased ROS levels in HEMn-LP cells; CBD significantly increased ROS by 29.29% at 1 µM and 24.90% at 2 µM (Figure 5A). THC increased ROS by 14.94% and 42.13% at 1 and 2 µM, respectively; however, the increase was statistically significant for THC at 2 µM and was also significant for THC (1 µM).

There was no change in ROS levels in HEMn-DP cells treated with either THC or CBD at any concentration (Figure 5B). Collectively, these results indicate that THC and CBD significantly increased oxidative-stress-related ROS generation in LP cells, while in DP cells, neither THC nor CBD had any effect on ROS levels.

## 4. Discussion

Results of this study demonstrate that (*i*) THC was less cytotoxic to both LP and DP cells as compared to CBD; THC appeared to show higher cytotoxicity to LP cells as compared to DP cells; (*ii*) THC as well as CBD exhibited a similar effect on the stimulation of melanin synthesis and dendricity in LP cells with full recovery of dendricity; (*iii*) THC and CBD showed similar effects of stimulating melanin synthesis in DP cells but showed marginal reduction in dendricity in DP cells; and (*iv*) THC and CBD induced ROS production in LP cells with no effect in DP cells. The results of significant cytotoxicity by exposure to 4 µM CBD for a duration of 6 d contrasts to a previous study [24] where authors reported no cytotoxicity to melanocytes exposed to CBD at 6 µM for 5 d; however, this difference might be due to experimental variability and origin of cells. The concentration ranges of THC and CBD selected in this study are well within those measured in the serum of cannabis users and have been used in previous studies [35,36].

We also conducted preliminary experiments to assess the role of CB_1_ and CB_2_ receptor on cellular melanin content by pharmacologically blocking CB_1_ and CB_2_ receptors using the selective CB_1_ receptor antagonist SR141716 and the selective CB_2_ receptor antagonist SR144528. Our results showed that in DP cells, the effects of increased melanin content by THC were partly rescued by CB_1_ receptor blockade (Figure S1A) but not by CB_2_ receptor blockade (Figure S1B), while unexpectedly, in the case of CBD, none of the receptor blockades had any effect (Figure S1C). These results indicate that in the case of THC, partial rescue of increased melanin content is attributable to the CB_1_ receptor which is in line with the reported partial agonist behavior of THC at CB_1_ and CB_2_ receptors [37]. Interestingly, the study by Hwang et al. [24], which does not specify cell pigmentation or donor type, showed the involvement of only the CB_1_ and not CB_2_ receptor in CBD-mediated increases in melanin synthesis in their cells, which contrasts with our results. Arguably, the differences are due to experimental variations and differences in cell donor used; this is especially evident since in the case of LP cells, we found that none of the receptor blockades could rescue increased melanin either by THC or by CBD (data not shown). Further in-depth studies are warranted to evaluate the donor-dependent effects on CB receptors as well as identify if other receptors such as transient receptor potential vanilloid 1 (TRPV 1) might have a role. In addition, the p38 MAPK pathway was previously shown to be involved in the melanogenesis-stimulating effects of CBD [24]; however, that pathway was not evaluated in our study and would be interesting for future investigation.

We did not conduct measurements of extracellular melanin in primary human melanocytes as they do not secrete any detectable amount of melanin into the culture medium; this has also been noted in previous studies by our group [38,39,40]. Dendricity is typically used as a surrogate metric for melanosome secretion; the dendrite length and number of dendrites were quantified to provide an accurate estimate of the effects of compounds on melanosome secretion. Previous studies conducted with CBD showed that increased melanin synthesis was correlated to increases in the protein levels and mRNA levels of MITF [24] although the authors did not report dendricity. The findings of increased dendricity in cells after exposure to THC and CBD might be attributed, at least in part, to increases in protein levels of microphthalmia transcription factor (MITF), a transcription factor involved in mediating melanosome export and dendricity [41,42]. A previous study by Hwang et al. [24] reported that the melanogenesis-stimulating effects of CBD in primary melanocytes were ascribed, in part, to an increase in the protein levels and mRNA expression of tyrosinase as well as other proteins MITF, TRP-1, and TRP-2 that are further involved in the melanogenesis pathway. We did not evaluate whether THC or CBD stimulated expression of tyrosinase or other proteins as it was not the focus of this pilot study. Further analysis of melanogenesis-regulating proteins such as tyrosinase as well as other proteins such as TRP-1, TRP-2, and MITF warrant future investigation. The dendrites of a melanocyte comprise actin and microtubule cytoskeletal elements that aid in the exportation of melanosomes [43]. In our study only the morphological changes of increased dendrite lengths in melanocytes after exposure to CBD or THC at nontoxic concentrations were reported and further confirmation on changes in cytoskeletal proteins such as actin or tubulin by immunocytochemistry were beyond the scope of this study and warrant future studies. We would like to highlight that our results of increased melanogenesis were accompanied by morphological changes in dendrites elongated by THC and CBD, which is reminiscent of the results of a previous study [44], where the authors reported similar findings in B16F10 melanoma cells that were treated with sub-cytotoxic concentrations of rhododenol, a phenolic compound, that was recalled in cosmetic industries due to melanocytotoxicity.

The TDL for the recovery control group was 38% higher than that of the exposure control group for LP cells. This can be explained as follows. We used low cell density during plating to avoid the entangling of cell dendrites with each other over the full duration of the experiments, which was 12 d, so that we could distinguish and measure TDL in our imaging software at the end of recovery. Due to this, during the exposure period of 6 d, cells were still in their proliferative phase, and in the recovery period of an additional 6 d, some cells were in the differentiating phase, due to which dendrite lengths increased. As we randomly sampled cells for our analysis and did not use any cut-off range on measured TDL, our TDL values were higher. Moreover, the study of the true recovery of dendrite lengths is challenging and there are no available protocols in the literature; in our previous study [39], we conducted a 3 d exposure with 9 d recovery study on darkly-pigmented cells, which do not typically show large increases in dendrite lengths unlike the effect observed in this study on lightly-pigmented cells.

The findings of higher levels of cellular ROS production by THC and CBD are in line with prior studies which showed that marijuana smoke [45,46,47] as well as the compounds THC [48] and CBD [49] can induce high levels of ROS in different cells, although to date, no study has reported ROS levels in melanocytes. Increased generation of ROS in skin has been shown to contribute to skin cancer [50]. Another study reported that oxidative stress due to higher ROS led to increases in levels of pheomelanin, the red-colored melanin polymer, in dysplastic nevi [51]. The basal ROS levels of LP and DP cells after 6 d treatment duration (as was used in our experiments) showed that ROS levels of LP cells were lower than that of DP cells (Figure S2). To the best of our knowledge, there is no study which reports the basal ROS levels of both LP and DP cells. Prior studies that evaluated ROS levels in melanocytes with different pigmentation contents only reported ROS levels after a short-term exposure to a natural or chemical stimulus. For example, a previous study reported that ROS levels of LP cells were higher as compared to DP cells after UVA radiation [52], although the authors did not report if the cells belonged to adult or neonatal donors. In another study [53], the ROS levels of melanocytes (that were isolated from multiple neonatal donors differing in melanin contents) were reported, but after 1 h treatment with a pro-oxidant cumene hydroperoxide the authors showed that ROS levels were inversely correlated to melanin content. In addition, another study [54] showed that melanocytes from Black and Caucasian skin donors did not have any difference in antioxidant activity levels of catalase (CAT), superoxide dismutase (SOD), peroxidase, and glutathione peroxidase (GPx) enzymes. Taken together, our results of higher ROS levels after a 6 d exposure to cannabinoids in LP cells as compared to DP cells are in agreement with the aforementioned studies that have also shown higher ROS generation in cells with lower melanin content after exposure to a chemical or natural stimulus. Since it has been shown that the ROS levels that are detected in melanocytes are dependent on the balance between the capacity of melanin to enhance the production of ROS [55,56] vs. its capacity to scavenge ROS [57], our results of higher basal ROS levels in DP cells as compared to LP cells might be attributed to that.

Previous studies that examined the impact of the tobacco xenobiotic, nicotine, on HEMn-LP and HEMn-DP cells, have evaluated antioxidant activities of CAT, SOD, and GPx enzymes [16,17]. Moreover, heme oxygenase -1 (HO-1) is also one of the primary antioxidant enzyme in melanocytes that acts through the nuclear factor-E2-related factor 2-antioxidant responsive element (Nrf2-ARE) pathway [58]. Examination of whether cannabinoids altered the antioxidant activity of melanocytes was beyond the scope of this study and, due to limitations, we did not conduct assays to evaluate the spectrum of the antioxidant activities of melanocytes. While our results demonstrate the induction of oxidative stress in melanocytes (by production of ROS), whether melanocytes are able to upregulate their antioxidant activities in response to the oxidative stress effectively or whether the antioxidant activities are exhausted warrants a thorough evaluation in a follow-up study along with the evaluation of the Nrf2 pathway, as both THC and CBD have been shown to induce HO-1 protein levels in human vascular smooth muscle cells [59] and CBD was shown to induce HO-1 in human keratinocytes [60].

The impact of THC on melanocyte biology is critical from a toxicological perspective since growing number of young adolescents, including experienced smokers, consume marijuana, which contains THC as the psychoactive component. THC is a lipophilic and neutral drug that has been reported to not bind strongly to the melanin pigment, unlike nicotine in tobacco products [61], although THC has been detected in the hair shafts of cannabis users [62]. Previous studies have documented the inhibitory effects of THC on melanin synthesis in human hair follicles [23] or human melanoma cell cultures [63]. The findings of the stimulation of melanin synthesis by THC cannot be compared to these prior studies as they did not use normal human epidermal melanocytes, although the results of stimulation of melanin content and tyrosinase activity of cells by CBD are in line with a previous report where CBD showed similar results at a higher concentration of 6 µM [24]. The source of melanocyte donor and skin phototype was not reported in these studies; thus, it is unknown if the effects would be observed in the same ethnicities. Caucasians are at a higher risk for skin cancer due to having less melanin [64] and melanocytes from light skin have been shown to be prone to the development of UVB-induced melanoma, but if their melanin synthesis capacity is stimulated due to exogenous chemical stimuli, these melanocytes also develop a high propensity for UVA-induced melanoma [65].

In summary, the results of this study demonstrate that LP cells were more sensitive to the biochemical effects of marijuana xenobiotics, THC and CBD, as compared to DP cells. THC and CBD exhibited similar effects in LP cells where both similarly increased melanin synthesis and melanin export (by selectively stimulating TDL over dendrite number, which was fully recovered upon the removal of the compounds), accompanied by significant ROS generation. In the case of DP cells, THC and CBD increased melanin synthesis in a similar fashion; however, no significant change was noted in dendricity. Although THC showed a marginal reduction in dendricity, no significance was achieved. Additionally, ROS levels were not altered compared to control for both compounds. These results highlight that the presence of higher melanin in dark-skinned individuals provides protection against the induction of oxidative stress [57,66], and that light-skinned individuals are at a higher incidence of increased pigmentation of skin coupled with higher oxidative stress when exposed to THC or CBD. This may be of concern, since LP melanocytes are prone to oxidative insult and exposure to THC or CBD may trigger an oxidative stress cascade. Although this study highlights the potential for the induction of adverse biochemical effects by THC and CBD, it is important to note that the study only examined a limited number of toxicological endpoints and did not examine the molecular mechanisms that underlie the observed biochemical effects. Further studies to expand upon the impact of THC and CBD on melanocyte functions and antioxidant status are warranted. Moreover, the effects of the combination of THC and CBD on melanocyte biology merits future investigation.

## Data Availability

The data presented in this study are available upon reasonable request from the corresponding author.

## Supplementary Materials

The following supporting information can be downloaded at: https://www.mdpi.com/article/10.3390/jox12020012/s1, Figure S1: Intracellular melanin levels of HEMn-DP cells treated with (A) THC and CB_1_ receptor antagonist; (B) THC and CB_2_ receptor antagonist and; (C) CBD with CB_1_ or CB_2_ receptor antagonist, for a duration of 6 d; in the figure axis CB_1_ and CB_2_ denote CB_1_ receptor antagonist SR141716, while CB_2_ denotes CB_2_ receptor antagonist SR144528, respectively, each used at a concentration of 0.2 µM. THC and CBD were used at a concentration of 2 µM. All data are mean ± SD of values combined from two independent experiments (n = 6); One-way ANOVA followed by Tukey’s test; [# *p* < 0.001 vs. Ctrl; * *p* < 0.05 vs. THC; ** *p* < 0.01 vs. Ctrl]. Cells treated with CB_1_ or CB_2_ receptor alone do not affect melanin content as expected; Figure S2: Basal ROS levels by DCFDA assay in DP cells and LP cells for a duration of 6 d and are expressed as RFU normalized to protein content. Data are mean ± SD of three independent experiments.
