# Supplementary material for: Comparative Study of Δ9-Tetrahydrocannabinol and Cannabidiol on Melanogenesis in Human Epidermal Melanocytes from Different Pigmentation Phototypes: A Pilot Study
Source: J Xenobiot. 2022 Jun 10;12(2):131–44. doi: 10.3390/jox12020012 (PMC9224588; doi:10.3390/jox12020012)
Supplement: Supplementary file 1 [file jox-12-00012-s001.zip › jox-1651702-supplementary.pdf]

Supplementary Information

Comparative study of Δ9-Tetrahydrocannabinol and Cannabidiol on Melanogenesis in Human Epidermal Melanocytes from Different Pigmentation Phototypes: A Pilot Study

Shilpi Goenka[1, 2*]

[1]Department of Biomedical Engineering, Stony Brook University, Stony Brook, NY 11794-5281, USA
[2] Department of Biochemistry and Cell Biology, Stony Brook University, Stony Brook, NY 11794-5215, USA
* Correspondence: shilpi.goenka@stonybrook.edu

Supplementary Method

HEMn-DP cells (1.1 × 10^5 cells/well) were cultured in 6-well plates for 72 h followed by the replacement of medium with THC or CBD (each at nontoxic concentration of 2 μM) in the presence or absence of CB_1 receptor antagonist SR141716 (99.2% purity, Axon Medchem, VA, USA) or CB_2 receptor antagonist SR144528 (99.8% purity, Axon Medchem, VA, USA) and further cultured for 6 d. At this point, melanin assay was conducted similar to methods reported in main text. Briefly, the cells were harvested, washed in PBS and 125 μL of 1N NaOH was added and heated to 70°C to solubilize melanin. Next, 100 μL aliquots of lysate were transferred to a 96-well plate and the absorbance was read at 475 nm using a microplate reader. The absorbance values were normalized to the total protein contents; Abs/ μg protein was expressed as percentage of control.

Supplementary Figures

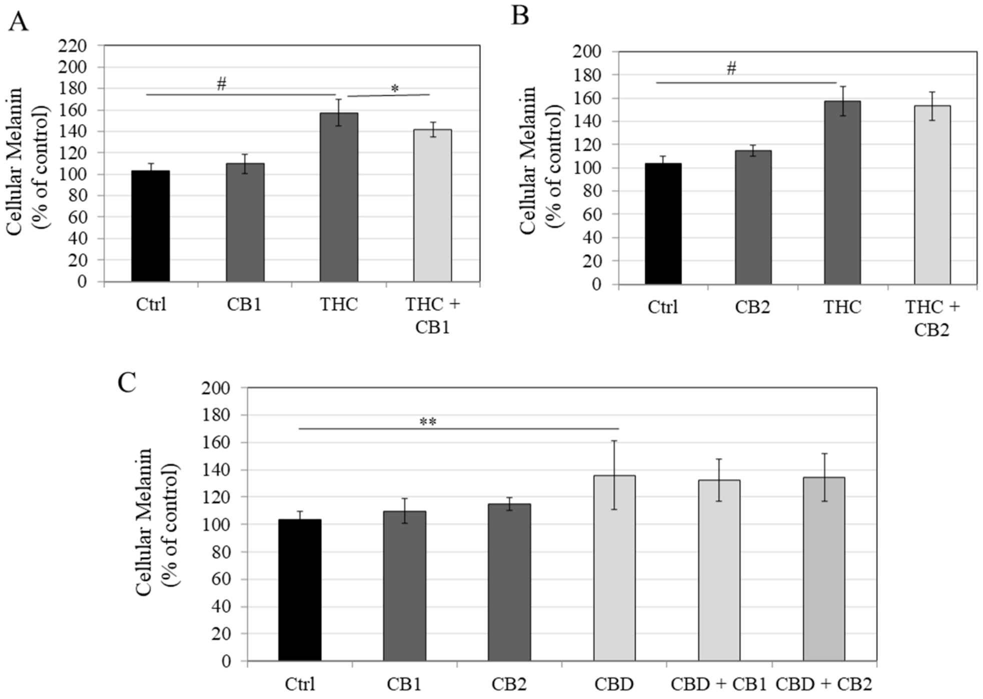

Figure S1. Intracellular melanin levels of HEMn-DP cells treated with (**A**) THC and CB_1 receptor antagonist; (**B**) THC and CB_2 receptor antagonist and; (**C**) CBD with CB_1 or CB_2 receptor antagonist, for a duration of 6 d; in the figure axis CB_1 and CB_2 denote CB_1 receptor antagonist SR141716, while CB_2 denotes CB_2 receptor antagonist SR144528, respectively, each used at a concentration of 0.2 μM. THC and CBD were used at a concentration of 2 μM. All data are mean ± SD of values combined from two independent experiments (n = 6); One-way ANOVA followed by Tukey's test; [#$p < 0.001$ vs. Ctrl; *$p < 0.05$ vs. THC; **$p < 0.01$ vs. Ctrl]. Cells treated with CB_1 or CB_2 receptor alone do not affect melanin content as expected.

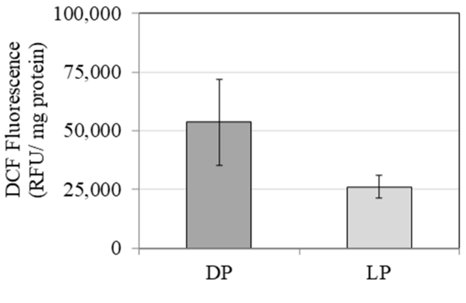

Figure S2. Basal ROS levels by DCFDA assay in DP cells and LP cells for a duration of 6 d and are expressed as RFU normalized to protein content. Data are mean ± SD of three independent experiments.
